# Characteristics and Biomarkers of Ferroptosis

**DOI:** 10.3389/fcell.2021.637162

**Published:** 2021-01-21

**Authors:** Xin Chen, Paul B. Comish, Daolin Tang, Rui Kang

**Affiliations:** ^1^Guangzhou Municipal and Guangdong Provincial Key Laboratory of Protein Modification and Degradation, The Third Affiliated Hospital, School of Basic Medical Sciences, Guangzhou Medical University, Guangzhou, China; ^2^Affiliated Cancer Hospital & Institute of Guangzhou Medical University, Guangzhou, China; ^3^Department of Surgery, UT Southwestern Medical Center, Dallas, TX, United States

**Keywords:** ferroptosis, cell death, biomarker, lipid perioxidation, iron metabolism

## Abstract

The induction and consequences of regulated cell death (RCD) are accompanied by changes in gene and protein expression, biochemical pathways, as well as cell morphology and size. Such RCDs have a significant impact on development, tissue homeostasis, and the occurrence and progression of disease. Among different forms of RCD, ferroptosis appears to be the main cause of tissue damage driven by iron overload and lipid peroxidation. In fact, the dysfunctional ferroptotic response is implicated in a variety of pathological conditions and diseases, such as neurodegenerative diseases, tissue ischemia-reperfusion injury, tumorigenesis, infections, and immune diseases. Ferroptotic response can be fine-tuned through various oxidative stress and antioxidant defense pathways, coupling with metabolism, gene transcription, and protein degradation machinery. Accordingly, a series of ferroptosis inducers or inhibitors targeting redox- or iron metabolism-related proteins or signal transduction have been developed. Although this kind of RCD has recently attracted great interest in basic and clinical research, detecting and monitoring a ferroptotic response still faces challenges. In this mini-review, we not only summarize the latest knowledge about the characteristics of ferroptosis *in vitro* and *in vivo*, but also discuss the specificity and limitations of current biomarkers of ferroptosis.

## Introduction

Cell death is a basic biological process that regulates cell fate, tissue regeneration, and the body’s immune response. Accidental cell death (ACD) and regulated cell death (RCD) are two major subcategories of cell death (Galluzzi et al., 2018). Unlike ACD, which is an uncontrolled rapid process, RCD usually utilizes clear molecular machinery involving multiple genes or proteins, and can be intervened on at multiple levels, especially using drugs or small molecular compounds (Tang et al., 2019). According to the publication record in PubMed,^1^ apoptosis, necroptosis, pyroptosis, and ferroptosis may be the most studied forms of RCD, involved in multiple pathological conditions and diseases. In some cases, several rare RCDs [e.g., alkaliptosis (Song et al., 2018b) and oxeiptosis (Holze et al., 2018)] have also been observed in cell cultures or mouse models, although their physiological or pathological significance remains largely unknown.

**FIGURE 1:**
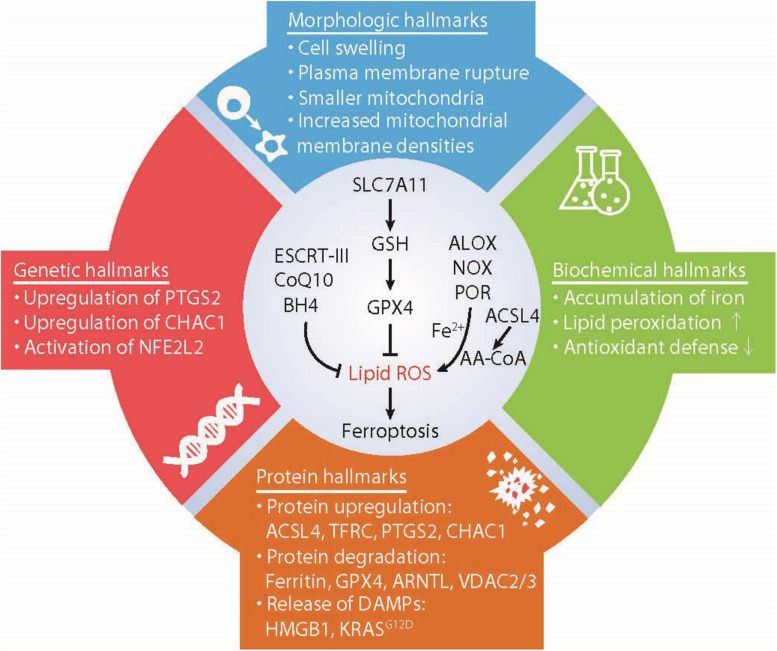
Hallmarks of ferroptosis. Ferroptosis is a type of iron-dependent regulated cell death mainly caused by unrestricted lipid peroxidation and subsequent membrane damage. Ferroptotic cell death may show some morphological and biochemical characteristics as well as common changes in gene and protein levels. AA-COA, arachidonoyl-coenzyme A; ACSL4, acyl-CoA synthetase long-chain family member 4; ALOX, lipoxygenases; ARNTL, aryl hydrocarbon receptor nuclear translocator like; BH4, tetrahydrobiopterin; CHAC1, ChaC glutathione specific gamma-glutamylcyclotransferase 1; COQ10, Coenzyme Q10; ESCRT-III, endosomal sorting complex required for transport-III; GPX4, glutathione peroxidase 4; GSH, glutathione; HMGB1, high mobility group box 1; NFE2L2, nuclear factor erythroid 2-like 2; NOX, NADPH oxidases; POR, cytochrome P450 oxidoreductase; PTGS2, prostaglandin-endoperoxide synthase 2; PUFA, polyunsaturated fatty acids; SLC7A11, solute carrier family 7 member 11; TFRC, transferrin receptor; VDAC2/3, voltage dependent anion channel 2/3.

Ferroptosis is a non-apoptotic and oxidative damage-related RCD (Dixon et al., 2012), mainly driven by iron accumulation, lipid peroxidation, and subsequent plasma membrane rupture (Tang and Kroemer, 2020). The process of ferroptosis is further controlled through a variety of molecular signaling achieved using epigenetic, transcription, and post-translational mechanisms (Chen et al., 2020b). After treatment with reagents or suffering environmental stresses, an increase in ferroptotic cell death can be observed in experimental models by activating the intrinsic or extrinsic pathways, which is discussed in depth in recent reviews (Chen et al., 2020b; Tang et al., 2020). Notably, two small molecular compounds, namely erastin (Dixon et al., 2012) and RSL3 (Yang et al., 2014), are the most commonly used reagents for triggering ferroptosis to study its molecular mechanisms. Excessive or deficient ferroptotic response also occurs in tissue samples of human diseases (Ashraf et al., 2020), which highlight the potential of pathological relevance of ferroptosis (Stockwell et al., 2017). In this min-review, we used some examples and their corresponding regulatory mechanisms to focus on the characteristics and biomarkers of ferroptosis (Figure 1).

## Morphological Hallmarks of Ferroptosis

What we call morphological features usually refers to cell changes observed through light or electron microscopy, and does not involve staining of special protein markers. Ferroptosis is generally a type of regulated necrosis, which is devoid of the morphological characteristics of apoptosis (e.g., cell shrinkage and plasma membrane blistering; Conrad et al., 2016). In contrast, necrotic morphology, such as cell enlargement and plasma membrane rupture, are commonly observed in most ferroptotic cells (Conrad et al., 2016). Consequently, the activation of cell membrane repair pathways, such as endosomal sorting complex required for transport-III (ESCRT-III) machinery, prevents ferroptotic cell death (Dai et al., 2020c). Electron microscopy shows that ferroptotic cells represent a vast change in mitochondrial ultrastructure, such as reduction in mitochondrial volume, increase in mitochondrial membrane density, and disappearance of mitochondrial cristae (Yagoda et al., 2007; Dixon et al., 2012). Since elevated autophagy promotes ferroptosis (Liu J. et al., 2020), autophagy-related ultrastructures (e.g., double-membrane autophagosomes and various lysosome-related vesicles) are often observed in ferroptotic cells or tissues (Friedmann Angeli et al., 2014). Although activators of ferroptosis lead to oxidative damage in DNA (Song et al., 2016), the nucleus of ferroptotic cells seem to be normal and lack chromatin concentration (Yagoda et al., 2007), which is a morphological marker of apoptotic cells. As a type of inflammatory RCD, immune cell infiltration is observed in tissues affected by the ferroptotic damage. For example, acute pancreatitis is a sterile inflammatory disease caused by the death of acinar cells. Ferroptotic acinar death contributes to experimental pancreatitis in mice, especially in cases of impaired circadian rhythms (Liu et al., 2020b). This ferroptosis-related pancreatitis is associated with pancreatic histological damage and leukocyte infiltration that can be detected using hematoxylin and eosin stain (Liu et al., 2020b). In general, the necrotic morphology caused by ferroptosis has been observed *in vitro* or in tissues, but it is difficult to distinguish ferroptosis from other types of regulated necrosis based on these changes alone.

## Biochemical Hallmarks of Ferroptosis

### Accumulation of Cellular Iron

Since the initial study defined ferroptosis as an iron-dependent RCD, iron accumulation-mediated biochemical events (e.g., elevated Fenton reaction or activated iron-containing enzymes) seem to be a biochemical sign of ferroptosis (Chen et al., 2020c). Iron is an essential trace element, distributed in various subcellular organelles (e.g., mitochondria and lysosomes). The level of intracellular or mitochondrial ferrous iron (Fe^2+^) is increased in ferroptotic cells or tissues, which can be monitored by a biochemical assay kit (Yuan et al., 2016a), Prussian blue staining (Lu et al., 2020), or probes [e.g., FerroFarRed (Homma et al., 2020) or Phen Green SK (Song et al., 2018a)]. In contrast, iron chelators (e.g., deferoxamine) limit ferroptosis *in vitro* (Dixon et al., 2012) or *in vivo* (Lu et al., 2020). The level of iron in cells is controlled by a complex network that involves the absorption, storage, utilization, and outflow of iron (Chen et al., 2020c). As expected, certain molecular regulators related to iron homeostasis control ferroptosis sensitivity. For example, iron uptake mediated by transferrin (TF; Gao et al., 2015), lactotransferrin (LTF; Wang et al., 2020), and transferrin receptor (TFRC; Gao et al., 2015), as well as nuclear receptor coactivator 4 (NCOA4)-dependent ferritin degradation (Hou et al., 2016) facilities ferroptotic cell death. It is worth noting that TFRC is considered as a biomarker of ferroptosis in cell cultures or tissues, and an anti-TFRC antibody (called 3F3-FMA) especially plays a role in indicating ferroptotic death or damage (Feng et al., 2020).

In contrast, heat shock protein family B small member 1 (HSPB1/HSP25/HSP27; Sun et al., 2015) or prominin-2 (PROM2; Brown et al., 2019) diminishes ferroptosis by preventing cytoskeleton-mediated iron uptake or promoting ferritin export to extracellular space, respectively. Moreover, iron chaperones, such as poly(RC) binding protein 1 (PCBP1), reduce ferroptosis sensitivity in hepatocytes (Protchenko et al., 2020). Whether different iron metabolism regulators play an equivalent role in ferroptosis is still inconclusive. More importantly, given the numerous types of iron-dependent enzymes present in different subcellular organelles, the process of enzyme coordination and their regulation of ferroptosis remain unclear.

### Induction of Lipid Peroxidation

Lipid peroxidation plays a central role in mediating ferroptosis (Yang and Stockwell, 2016). In particular, the oxidation of polyunsaturated fatty acids (PUFAs) by reactive oxygen species (ROS) to produce lipid hydroperoxides is the most important hallmark of ferroptosis (Kuang et al., 2020). In addition to iron-triggered ROS production by the Fenton reaction, mitochondria- or NADPH oxidase (NOX)-mediated ROS production (Dixon et al., 2012; Gao et al., 2015; Xie et al., 2017; Yang W. H. et al., 2019) also play a cell type-dependent role in initiating lipid peroxidation. The key enzymes of lipid peroxidation that causes ferroptosis are the isoforms of arachidonate lipoxygenase (ALOX), including ALOX5, ALOX12, ALOX15, ALOX15B, and ALOXE3 in humans (Yang et al., 2016; Wenzel et al., 2017; Chu et al., 2019; Li et al., 2020). Alternatively, cytochrome P450 oxidoreductase (POR) may mediate lipid peroxidation in an ALOX-independent manner (Zou et al., 2020).

The production of PUFA for subsequent lipid peroxidation requires the activation of several upstream lipid synthesis and metabolism pathways, especially acyl-CoA synthetase long-chain family member 4 (ACSL4)-mediated conversion of arachidonic acid (AA) to AA-CoA (Yuan et al., 2016b; Doll et al., 2017; Kagan et al., 2017). Critically, the up-regulation of ACSL4 expression, but not the expression of other ACSL members, is a biomarker indicative of ferroptosis sensitivity (Yuan et al., 2016b). While acyl-CoA synthetase long-chain family member 3 (ACSL3) is not a biomarker of ferroptosis, ACSL3-mediated monounsaturated fatty acids (MUFAs) production limits oxidative PUFA-mediated ferroptosis in certain cancer cells (Magtanong et al., 2019), indicating that ACSL4- or ACSL3-dependent fatty acid metabolism plays opposite roles in ferroptosis.

Several assays can be used to detect and quantitate the extent of lipid peroxidation of ferroptotic cells *in vitro* and *in vivo*. First, C11 BODIPY 581/591 (Dixon et al., 2012) and LiperFluo (Kagan et al., 2017) are lipid-soluble fluorescent probes, which are widely used to monitor lipid peroxidation in cell cultures by microscope, plate reader, or flow cytometry. Functionally, LiperFluo is better than C11-BODIPY in indicating lipid peroxidation during ferroptosis, because it directly interacts with (phosphate) lipid hydroperoxide (Kagan et al., 2017). Second, liquid chromatography with tandem mass spectrometry (LC-MS/MS) analysis may be used to analyze changes in lipid profile, quantitative lipid peroxide species (PUFA-OOH and PL-OOH) or identification of oxidative proteins in the process of ferroptosis (Kagan et al., 2017). Third, through biochemical, enzyme-linked immunosorbent assay (ELISA) or staining methods, the detection of the final product of lipid peroxidation [e.g., malonyl dialdehyde (MDA; Ye et al., 2020) and 4-hydroxynon-enal (4-HNE; Shintoku et al., 2017)] or oxidative DNA damage biomarkers [e.g., phosphorylated H2A.X variant histone (γH2AX; Song et al., 2016) and 8-hydroxydeoxyguanosine (8-OH-dG; Zhang et al., 2019; Dai et al., 2020b)] has been applied in various samples *in vitro* or *in vivo*. However, the specificity and sensitivity of each measurement are different, which may affect the interpretation of the results.

### Loss of Antioxidant Defense

In support of the role of lipid peroxidation in ferroptosis, the classic ferroptosis inducers, such as erastin (Dixon et al., 2012) and RSL3 (Yang et al., 2014), are indeed inhibitors of the antioxidant system. The three antioxidant defense systems [referred to as glutathione (GSH; Dixon et al., 2012), coenzyme Q10 (CoQ10; Bersuker et al., 2019; Doll et al., 2019), or tetrahydrobiopterin (BH4) system (Kraft et al., 2020; Soula et al., 2020)] can work together or separately to limit ferroptotic death mediated by oxidative damage. The GSH system is the main pathway limiting ferroptosis. The inhibition of the upstream regulator system xc^–^ (an amino acid antiporter) or the downstream effector glutathione peroxidase 4 (GPX4) of GSH by drugs or small molecule compounds is recognized as the classic external or internal pathway of ferroptosis (Tang and Kroemer, 2020). In addition to blocking the system xc^–^ activity on the cell membrane, erastin is also a potential activator of mitochondrial voltage dependent anion channel 2/3 (VDAC2/3) (Yagoda et al., 2007), highlighting the participation of mitochondria dysfunction in erastin-induced ferroptosis. GPX4-independent anti-ferroptosis pathway relies on the production of CoQ10 (Bersuker et al., 2019; Doll et al., 2019) or BH4 (Kraft et al., 2020; Soula et al., 2020), which is further regulated by apoptosis inducing factor mitochondria associated 2 (AIFM2/FSP1) or GTP cyclohydrolase 1 (GCH1), respectively. Interestingly, AIFM2 was previously thought to be a pro-apoptotic protein in mitochondria (Wu et al., 2002). In contrast, the translocation of AIFM2 from the mitochondria to the membrane converts this pro-apoptotic activity into an anti-ferroptotic effect (Bersuker et al., 2019; Doll et al., 2019). The location and function switch of AIFM2 from apoptosis to ferroptosis depends on its post-translational modification, such as myristoylation (Bersuker et al., 2019; Doll et al., 2019). Other studies have shown that the anti-ferroptotic effect of AIFM2 may depend on its function of promoting membrane repair, rather than the production of reduced CoQ10 (Dai et al., 2020d). Since GSH, CoQ10, and BH4 are broad-spectrum antioxidants, these studies have also raised questions about the specific molecular mechanisms of ferroptosis. Indeed, conditional depletion of GPX4 in mice not only mediates ferroptosis (Friedmann Angeli et al., 2014), but also triggers apoptosis (Ran et al., 2003), necroptosis (Canli et al., 2016), or pyroptosis (Kang et al., 2018). Therefore, testing the levels of these antioxidants alone may not be enough to indicate changes in ferroptosis levels.

## Genetic Hallmarks of Ferroptosis

### Upregulation of *PTGS2* Gene

The well-known function of prostaglandin-endoperoxide synthase 2 (PTGS2/COX2) is to metabolize AA into prostaglandins. *PTGS2* is the most upregulated gene among 83 oxidative stress-associated genes in BJeLR cells following treatment with erastin or RSL3 (Yang et al., 2014). The up-regulation of *PTGS2* mRNA is used as a pharmacodynamic marker of ferroptotic tissues in mice exposed to erastin or RSL3 (Yang et al., 2014; Sun et al., 2015). Although it is a widely used biomarker of ferroptosis *in vitro* or *in vivo*, PTGS2 inhibitor (e.g., indomethacin) fails to affect ferroptotic cell death (Yang et al., 2014), indicating it is not a contributor of ferroptosis. In contradiction, *MIR212*-mediated the downregulation of *PTGS2* mRNA prevents ferroptotic neuronal death in a traumatic brain injury mouse model (Xiao et al., 2019), suggesting a cell type-dependent role of PTGS2 in ferroptosis. Further mechanism studies suggest that the up-regulation of *PTGS2* gene expression in ferroptosis requires lipid peroxidation, because antioxidant vitamin E or toxic 4-HNE can inhibit or induce PTGS2 expression in cancer cells or macrophages, respectively (Kumagai et al., 2004; Yang et al., 2014). The great challenge of PTGS2 as a biomarker of ferroptosis is that the up-regulation of PTGS2 is observed under various inflammatory conditions (FitzGerald, 2003), at least some of which are non-ferroptotic conditions.

### Upregulation of *CHAC1* Gene

ChaC glutathione specific gamma-glutamylcyclotransferase 1 (CHAC1/BOTCH) has γ-glutamyl cyclotransferase activity and reduces intracellular GSH levels by digesting glutathione into 5-oxoproline and cysteinylglycine dipeptide (Kumar et al., 2012). RNA sequencing studies show that *CHAC1* is the most up-regulated gene after treatment with systemic xc^–^ inhibitors (e.g., erastin and sorafenib) *in vitro* (Dixon et al., 2014). Later, the upregulation of *CHAC1* mRNA is confirmed in tissues from mice treated with certain ferroptosis inducers (e.g., erastin and artesunate; Xie et al., 2017; Wang N. et al., 2019). However, CHAC1 is not a biomarker of ferroptosis caused by the GPX4 inhibitor RSL3 or the GSH synthesis inhibitor buthionine sulphoximine (Dixon et al., 2014). Further functional studies have shown that CHAC1-mediated GSH degradation acts as a promoter of ferroptosis induced by erastin or artesunate, which is downstream of the activation of the endoplasmic reticulum (ER) stress pathway [especially the eukaryotic translation initiation factor 2 alpha (EIF2A)-activating transcription factor 4 (ATF4) pathways] (Mungrue et al., 2009; Dixon et al., 2014; Wang N. et al., 2019). Therefore, the upregulation of *CHAC1* gene expression provides a selective pharmacodynamic marker for ferroptosis induced by system xc^–^ inhibitors.

### Activation of *NFE2L2* Targeted Genes

The nuclear factor erythroid 2-like 2 (NFE2L2/NRF2) is a key transcription factor for cell survival during oxidative stress by activating the expression of detoxification and antioxidant genes. While the activation of the NFE2L2 pathway relies on the inhibition of the kelch like ECH associated protein 1 (KEAP1)-mediated degradation by ubiquitin-proteasome system (UPS) pathway, the excessive up-regulation of the NFE2L2 target gene may reflect the increase in oxidative damage during the activation of ferroptosis. This notion was first described in sorafenib-induced ferroptosis in hepatocellular carcinoma cells identifying metallothionein 1 G (MT1G) as a new NFE2L2-target gene responsible for ferroptosis resistance (Sun et al., 2016a,b). Increasing evidence points out that NFE2L2 plays a critical role in protecting damage under various ferroptotic conditions *in vitro* or *in vivo*. Many NFE2L2 targeted genes are upregulated in ferroptosis and these genes are involved in iron metabolism [e.g., ferritin heavy chain 1 (FTH1; Sun et al., 2016b; Shin et al., 2018), solute carrier family 40 member 1 (*SLC40A1*; Chang et al., 2018; Shin et al., 2018), heme oxygenase 1 (HMOX1; Sun et al., 2016b; Chang et al., 2018; Shin et al., 2018; Fang et al., 2019), and *MT1G* (Sun et al., 2016a)], GSH metabolism [e.g., solute carrier family 7 member 11 (*SLC7A11*; Chen et al., 2017), cystathionine beta-synthase (*CBS*; Liu N. et al., 2020), *CHAC1* (Gagliardi et al., 2019), and ATP binding cassette subfamily C member 1 (*ABCC1/MRP1*; Cao et al., 2019)], and detoxification or antioxidant responses [e.g., NAD(P)H quinone dehydrogenase 1 (NQO1; Sun et al., 2016b; Telorack et al., 2016; Shin et al., 2018), thioredoxin reductase 1 (*TXNRD1*; Shin et al., 2018; Takahashi et al., 2020), aldo-keto reductase family 1 member C1/2/3 (*AKR1C1/2/3*; Gagliardi et al., 2019)]. However, it is difficult to distinguish the role of NFE2L2 in ferroptotic- and non-ferroptotic RCD if only relying on the detection of the expression of NFE2L2 target gene.

## Protein Hallmarks of Ferroptosis

### Protein Upregulation

Genes are used to guide protein synthesis. Thus, the proteins corresponding to the genes mentioned above can theoretically be used to evaluate the sensitivity to ferroptosis. Western blot analysis of ACSL4, TFRC, PTGS2, or CHAC1 protein expression and immunohistochemical or immunofluorescence analysis of their signal or location distribution has been used to monitor ferroptotic response *in vitro* and/or *in vivo*. Since excessive autophagy promotes ferroptosis, the detection of the protein conversion of microtubule associated protein 1 light chain 3 (MAP1LC3)-I to MAP1LC3-II may reflect the degree of damage caused by ferroptosis activators (Zhou et al., 2020). Combining the use of various lysosomal inhibitors (e.g., chloroquine) to analyze changes in autophagic flux through various protein probes (e.g., RFP-GFP-LC3B or GFP-LC3-RFP-LC3ΔG) is also a commonly used detection method *in vitro* (Sun et al., 2018; Li et al., 2020).

### Protein Degradation

In addition to control by gene transcription, the intracellular level of protein is affected by protein degradation. Both UPS and autophagy pathway participate in the regulation of ferroptosis sensitivity in a context-dependent manner. In particular, autophagic degradation of anti-ferroptotic protein appears to be a good indicator of ferroptosis sensitivity. This type of autophagy-dependent ferroptosis was first described in erastin-induced ferritin degradation in mouse embryonic fibroblasts and pancreatic ductal adenocarcinoma (PDAC) cells (Hou et al., 2016). Ferritinophagy, namely autophagic degradation of ferritin, is mediated by the cargo receptor NCOA4 (Gao et al., 2016; Hou et al., 2016), which may be a useful marker for this process. The degradation of GPX4 protein can also be observed in ferroptosis-sensitive cells in response to various reagents, such as erastin (Zhu et al., 2017; Wu et al., 2019), RSL3 (Liu et al., 2020a), FIN56 (Shimada et al., 2016), and PdPT (Yang L. et al., 2020). The ER molecular chaperone heat shock protein family A (hsp70) member 5 (HSPA5) prevents GPX4 degradation in PDAC cells by the protein-protein interaction (Zhu et al., 2017). In contrast, heat shock protein 90 (HSP90)-dependent chaperone-mediated autophagy (CMA) promotes GPX4 degradation in neuronal cells or breast cancer cells (Wu et al., 2019). In addition to autophagy, UPS also mediates GPX4 degradation, but the mechanism remains unclear (Yang L. et al., 2020). Clockophagy, a type of selective autophagy for the degradation of clock circadian regulator aryl hydrocarbon receptor nuclear translocator like protein 1 (ARNTL/BMAL1) through sequestosome 1 (SQSTM1/p62), promotes ferroptosis by inhibiting hypoxia inducible factor 1 subunit alpha (HIF1A)-dependent lipid uptake and storage (Yang M. et al., 2019). On the contrary, UPS-mediated degradation of VDAC2/3 may limit the anticancer activity of erastin in melanoma cells (Yang Y. et al., 2020). These findings indicate that protein degradation pathways play a dual role in determining the sensitivity of ferroptosis, relying on its degrading substrates.

### Protein Release

The immune characteristics of cell death are not only factors affecting the occurrence and development of inflammatory diseases, but also involved in the regulation of tumor immunity (Galluzzi et al., 2020). This process usually depends on the release of damage associated molecular patterns (DAMPs) and subsequent activation of DAMP receptors. DAMPs are endogenous molecules, including protein and non-protein subgroups (Tang et al., 2012). High mobility group box 1 (HMGB1) is a typical nuclear DAMP (Kang et al., 2014), which triggers an immune response during various types of RCD, including ferroptosis (Wen et al., 2019). Advanced glycosylation end-product specific receptor (AGER/RAGE) has been recognized as a receptor for HMGB1, responsible for the inflammatory response caused by ferroptotic cell death in macrophages (Wen et al., 2019). Another protein-related DAMP marker involved in ferroptosis includes mutated KRAS protein (KRAS^*G*12*D*^) (Dai et al., 2020a). The release of KRAS^*G*12*D*^ by ferroptotic cancer cells is then taken up by macrophages through its receptor AGER (Dai et al., 2020a). This cell–cell communication results in M2 macrophage polarization and subsequent tumor formation in xenograft models (Dai et al., 2020a). Conversely, ferroptotic cell death may promote anti-tumor immunity by activating cytotoxic T cell responses (Wang W. et al., 2019), although the key DAMP mediator of this process is unidentified. There are still many uncertainties in the interaction of protein and non-protein (e.g., oxidative lipid or host DNA) DAMPs in shaping ferroptosis-associated inflammation and immune response, including their receptors and target immune cells.

## Ferroptosis in Diseases

Ferroptosis is implicated in many pathological conditions of iron overload, including cancer (Chen et al., 2020a; Conrad et al., 2020; Tang et al., 2020). On the one hand, ferroptosis caused by small molecule compounds can inhibit tumor growth in mouse models. For example, in a genetically engineered mouse model of pancreatic cancer, pancreatic tumors treated with cyst(e)inase show a ferroptotic morphological phenotype, with mitochondrial defects and extensive lipid droplet formation (Badgley et al., 2020), which may act as a source of PUFA for lipid peroxidation (Bai et al., 2019). Immunohistochemical staining of 4-HNE further indicates that the level of lipid peroxidation in pancreatic tumors is increased (Badgley et al., 2020). On the other hand, DAMP released by ferroptotic cells can promote tumor growth by maintaining an immunosuppressive microenvironment. For example, conditional depletion of pancreatic *Gpx4* or a high-iron diet triggers the release of mutant KRAS^*G*12*D*^ protein or nuclear DNA, thereby inducing the tumor-promoting effects of macrophages (Dai et al., 2020a,b). The expression level of KRAS^*G*12*D*^ in macrophages is negatively correlated with the survival of pancreatic cancer patients (Dai et al., 2020a). Therefore, these findings suggest that the simultaneous detection of intracellular and extracellular markers may help diagnose and treat diseases related to ferroptotic damage.

## Conclusion and Perspectives

In the past several years, we have witnessed the rapid development of ferroptosis research. This trend provides more opportunities to think deeply about the differences in the molecular mechanisms of RCD. A key unresolved question is how ferroptotic response causes cell death. Although the onset and intermediate signals and processes of ferroptosis have been described, the executioner of ferroptosis is still unknown. Unfortunately, the biomarkers of ferroptosis discussed in this review also present in other types of RCD or pathological conditions. A more precise understanding of specific biomarkers and contributors of ferroptosis (not non-ferroptotic deaths) may provide new opportunities for designing treatments for iron overload-related diseases. A combination of multiple biomarkers may help detect ferroptotic cell death in time. The challenge remains how to transform basic research findings into clinical applications. Solving these challenges requires further understanding of the molecular mechanisms and signal transduction of ferroptosis, as well as the use of new technologies to discover specific biomarkers.

## Author Contributions

XC, RK, and DT conceived the topic for this review. All authors listed have made a substantial, direct and intellectual contribution to the work, and approved it for publication.

## Conflict of Interest

The authors declare that the research was conducted in the absence of any commercial or financial relationships that could be construed as a potential conflict of interest.

## Footnotes

1 https://pubmed.ncbi.nlm.nih.gov/
